# Acute HIV infection detection and immediate treatment estimated to reduce transmission by 89% among men who have sex with men in Bangkok

**DOI:** 10.7448/IAS.20.1.21708

**Published:** 2017-06-28

**Authors:** Eugène D.M.B. Kroon, Nittaya Phanuphak, Andrew J. Shattock, James L.K. Fletcher, Suteeraporn Pinyakorn, Nitiya Chomchey, Siriwat Akapirat, Mark S. de Souza, Merlin L. Robb, Jerome H. Kim, Frits van Griensven, Jintanat Ananworanich, David P. Wilson

**Affiliations:** ^a^ Thai Red Cross AIDS Research Centre, Bangkok, Thailand; ^b^ SEARCH, Bangkok, Thailand; ^c^ Kirby Institute, University of New South Wales, Sydney, Australia; ^d^ US Military HIV Research Program (MHRP), Walter Reed Army Institute of Research, The Henry M. Jackson Foundation for the Advancement of Military Medicine, Bethesda, MD, USA; ^e^ Department of Retrovirology, US Army, Medical Component, Armed Forces Research Institute of Medical Sciences, Bangkok, Thailand; ^f^ International Vaccine Institute, Seoul, South Korea; ^g^ Department of Preventive Medicine and Public Health, University of California at San Francisco, San Francisco, CA, USA; ^h^ Burnet Institute, Melbourne, Australia

**Keywords:** HIV, acute infection, men who have sex with men, models/projections, prevention of sexual transmission, behavioural interventions, antiretroviral therapy

## Abstract

**Introduction**: Antiretroviral treatment (ART) reduces HIV transmission. Despite increased ART coverage, incidence remains high among men who have sex with men (MSM) in many places. Acute HIV infection (AHI) is characterized by high viral replication and increased infectiousness. We estimated the feasible reduction in transmission by targeting MSM with AHI for early ART.

**Methods**: We recruited a cohort of 88 MSM with AHI in Bangkok, Thailand, who initiated ART immediately. A risk calculator based on viral load and reported behaviour, calibrated to Thai epidemiological data, was applied to estimate the number of onwards transmissions. This was compared with the expected number without early interventions.

**Results**: Forty of the MSM were in 4th-generation AHI stages 1 and 2 (4thG stage 1, HIV nucleic acid testing (NAT)+/4thG immunoassay (IA)-/3rdG IA–; 4thG stage 2, NAT+/4thG IA+/3rdG IA–) while 48 tested positive on third-generation IA but had negative or indeterminate western blot (4thG stage 3). Mean plasma HIV RNA was 5.62 log^10^ copies/ml. Any condomless sex in the four months preceding the study was reported by 83.7%, but decreased to 21.2% by 24 weeks on ART. After ART, 48/88 (54.6%) attained HIV RNA <50 copies/ml by week 8, increasing to 78/87 (89.7%), and 64/66 (97%) at weeks 24 and 48, respectively. The estimated number of onwards transmissions in the first year of infection would have been 27.3 (95% credible interval: 21.7–35.3) with no intervention, 8.3 (6.4–11.2) with post-diagnosis behaviour change only, 5.9 (4.4–7.9) with viral load reduction only and 3.1 (2.4–4.3) with both. The latter was associated with an 88.7% (83.8–91.1%) reduction in transmission.

**Conclusions**: Disproportionate HIV transmission occurs during AHI. Diagnosis of AHI with early ART initiation can substantially reduce onwards transmission.

## Introduction

Antiretroviral treatment (ART) reduces HIV viral load and HIV transmission in HIV-discordant heterosexual couples [1–6]. However, despite increased uptake of ART since 1996, HIV incidence continues to rise among men who have sex with men (MSM) globally [7,8]. Fisher and colleagues reported a longitudinal phylogenetic analysis of HIV-1 in a UK MSM cohort from a population with a rising HIV epidemic in which onwards HIV transmission is significantly associated with recent infection, and the majority of new infections appear to occur from individuals who were as yet undiagnosed, and thus prior to initiating ART [9]. These findings are confirmed by multiple phylogenetic studies in MSM cohorts in Denmark, Switzerland, Canada, the USA and China [10–16]. While such phylogenetic studies are lacking in the Thai MSM population, newly HIV-infected MSM in Bangkok, Thailand, are mostly young men connected to circles of highly sexually active young MSM in a multi-risk factor environment, including high partner turnover, stimulant drug use, concurrent sexually transmitted infections (STIs) and unidentified newly HIV-infected other MSM [17].

Acute HIV infection (AHI) is characterized by high viral replication in blood and genital secretions and hence increased infectiousness to which characteristics of founder viruses may also contribute [18–26]. Here, we describe observed reductions in HIV-1 plasma viral load and transmission risk behaviour in a cohort of acutely infected Thai MSM. We use an HIV risk calculator model, calibrated to reflect HIV incidence and prevalence among Thai MSM and informed by published data on the relationship between viral load and transmission risk and observed data from the cohort, to estimate the impact of viral load control and behavioural change on epidemic spread through interventions targeting Thai MSM at high risk.

## Methods

### Study design

Study RV254/SEARCH 010 is a prospective cohort study conducted in Bangkok, Thailand, which enrols predominantly Thai participants in the earliest stages of HIV-1 infection. AHI staging was based on a combination of HIV RNA detection (nucleic acid testing (NAT) testing) 4th-generation (4thG) immunoassay (IA), third-generation (3rdG) IA and western blot results: 4thG AHI stages 1, 2 and 3 (4thG stage 1, NAT+/4thG IA-/3rdG IA–; 4thG stage 2, NAT+/4thG IA+/3rdG IA–; 4thG stage 3, NAT+/4thG IA+/3rdG IA+/western blot– or indeterminate) [27]. Both 4thG and 3rdG IAs detect IgM, but the 4thG assay also detects p24 antigen. Between April 2009 and March 2013, 69,911 samples were screened to identify 112 acutely infected participants and 100 enrolled: 90 MSM, 8 heterosexual women and 2 heterosexual men. Two of the MSM opted to not start ART immediately. Here we include data from the 88 MSM enrolled and treated with immediate ART, all of whom had reached beyond 24 weeks on study at time of analysis. Clinical, immunological and virological characteristics of the study participants are primary endpoints and have been described in several publications [27–32]. The study also collects descriptive data of demographics and behavioural risk, and characteristics of sexual contacts. Study interventions include immediate ART upon diagnosis and behavioural counselling. Outcomes measured are markers of disease progression, including CD4+ T cell count, HIV RNA in blood and behavioural changes. Plasma HIV RNA data and behavioural data were used in a risk calculator model (described below) to estimate the number of onwards transmissions averted from this cohort due to detection of AHI, behaviour change and ART initiation. The study is registered at clinicaltrials.gov (NCT00796146) and is approved by the institutional review boards (IRBs) of Chulalongkorn University, Thailand, and Walter Reed Army Institute of Research, USA. All participants gave informed consent. Participants who elected to start immediate ART were co-enrolled in a local protocol (clinicaltrials.gov: NCT00796263) approved by the Chulalongkorn University IRB.

### Setting and participants

Participants who presented for voluntary HIV counselling and testing (VCT) with 4thG IA at two sites in Bangkok, Thailand (the Thai Red Cross Anonymous Clinic and Silom Community Clinic) and gave informed consent had blood samples screened for AHI by pooled HIV NAT and sequential IA according to published methods [27,32]. AHI was defined as either being 4thG IA positive with positive HIV RNA and negative or indeterminate western blot, or 4thG IA negative while testing positive for HIV RNA by both qualitative (NAT) and quantitative (HIV RNA) assays. Any VCT client aged 18 years or older with confirmed AHI is eligible for study RV254/SEARCH 010 screening. ART for all participants opting for immediate treatment includes efavirenz, tenofovir and emtricitabine or lamivudine, with or without raltegravir and maraviroc. Substitution with an integrase or protease inhibitor is used for participants with efavirenz intolerance.

### Laboratory methods

HIV RNA was measured in plasma at baseline and weeks 2, 4, 8, 12, 16, 20, 24 and every three months thereafter by either the COBAS® AMPLICOR HIV-1 Monitor Test v1.5 (Roche Molecular Systems, Inc., Pleasanton, CA, USA) or the COBAS®AmpliPrep/COBAS® TaqMan® HIV-1 test v2.0 (Roche Molecular Systems, Inc., Pleasanton, CA, USA) with analyses based on lower limit of detection of 50 copies/ml.

### Measures

The number and type of sexual partners (steady, casual), condom use and specific sexual behaviours were assessed by standardized written questionnaires asking quantifying information which were completed by participants in a private setting at baseline, week 24 and week 48. . The questionnaire asked data about number of partners; oral/anal intercourse practices, role (receptive, insertive or both) and condom use; and knowledge about HIV sero-status of each partner in the preceding four months.

Questions regarding behavioural practices, such as unprotected anal intercourse, were asked as part of standard medical history from each participant by the study physicians at each visit. If crucial information was missing from either questionnaire or medical history, these were supplemented by each other in the final data set used for this analysis.

Estimated date of infection was assessed from questionnaire and medical history.

### Statistical and risk calculation methods

Study participants who reported sex with male partners and opted for immediate ART were included in the analysis. Statistical analyses were done using Stata/IC 12.1 for windows (StataCorp LP, College Station, TX, USA).

### Risk calculator model methods

**Figure 1.**
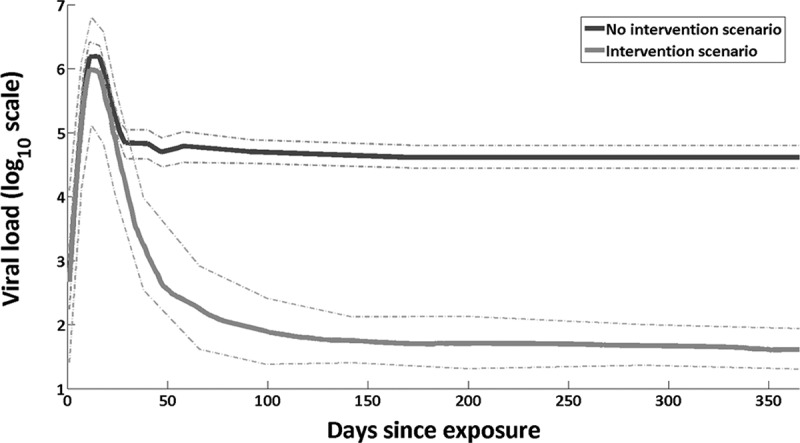
Viral load trajectory [33].

Time-dependent individual-level plasma HIV viral load and reported transmission-related behaviour data were used to inform a risk-equation model. The model included the (viral load-dependent) transmission probability per-act and frequency of types of transmission-related acts. Calculations were calibrated by adjustment of the parameter for transmission probability per-act associated with viral load set point to reflect HIV incidence estimates among MSM in Bangkok of 5.9 infections per 100 person years (95% CI 5.2–6.8) and an HIV prevalence estimate of 23.9% (95% CI 21.1–27.0) [17]. This model was then used to estimate the number of new HIV infections caused by each person in the first year after exposure. Two main scenarios were projected: (i) a baseline scenario which incorporated the interventions as reported in the data and (ii) a hypothetical scenario with no early detection and treatment. Under the hypothesized scenario of no early detection and treatment, the viral load of each individual was assumed to follow a standard trajectory, see Figure 1 [33].

Transmission risk behaviours of the cohort reported at the time of diagnosis were kept constant across the simulation in this counterfactual/no-intervention scenario. Two further hypothetical scenarios were simulated: (i) a scenario where only behavioural changes were considered as reported in the cohort data and (ii) a scenario in which behaviours were assumed to be as they were just prior to diagnosis but viral load reduction was considered as measured in the study. These scenarios were projected to distinguish between the influence of viral load reduction through treatment and diagnosis-related risk behaviour changes.

We used an adaptation of Røttingen and Garnett’s previously reported equation 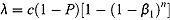, which estimates the number of onwards infections caused by an average infected individual over a given period of time [34]. Here, 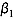 is the transmissibility of one unprotected act between two individuals and 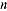 is the number of risk acts between the individuals, 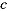 is the number of partners of an average infected individual and 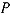 is the prevalence of infection among the population; hence 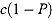 represents the number of contacts without HIV. The use of condoms and different forms of sexual contact and associated risk behaviours can then be incorporated into this base equation, leading to

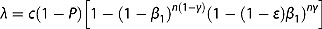

where 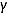 is the proportion of sexual acts protected by a condom and 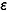 is the efficiency of condom use in reducing transmissibility.

There is a strong relationship between HIV viral load and transmission [35,36]. Blaser et al. recently conducted a meta-analysis of estimates around this relationship and found a 2.09-fold (95% CI 1.47–2.97) increase in infectiousness per 10-fold increase in viral load amongst discordant heterosexual couples [36]. Using this relationship, we estimate the transmissibility per average unprotected act involving an average infected individual to be

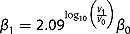

where 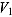 is the viral load during any particular risk event and 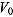 is the average baseline viral load corresponding to a baseline transmission probability (we take 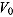 to be the viral load set point among untreated individuals and 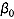 is calibrated to reflect the incidence of HIV among MSM in Bangkok) [17,33]. The calculated value of 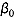 was found to be 0.67 (0.59–0.76). This is in reasonable agreement with independent pooled estimates of transmission risks calculated by separate meta-analyses for anal intercourse [37,38].

## Results

### Data collection

Baseline characteristics of the 88 participants are summarized in Table 1. At time of analysis all 88 had reached 24 weeks on study while 66/88 had reached 48 weeks on study. Mean age at time of AHI was 28.5 years and mean estimated duration of infection was 16.5 days. The majority of participants were in 4thG stage 3 (*n* = 48), the remainder in 4thG stages 1 (*n* = 19) and 2 (*n* = 21). Mean CD4+T cell count was 412 cells/mm^3^, mean plasma HIV RNA 5.62 log_10_ copies/ml and the majority of participants (73%) were infected with HIV-1 subtype CRF_01AE, the predominant circulating form in Thai MSM [39]. Completed questionnaire data were available for 86 participants at baseline.

**Table 1.** Baseline characteristics

Baseline characteristics of 88 MSM	Total (*n* = 88)
Age (years), mean (SD)	28.5 (7.0)
4thG stage^a^, *n* (%)	
1	19 (21.6)
2	21 (23.9)
3	48 (54.6)
Infection duration (days), mean (SD)	16.5 (6.6)
CD4T cell count (cells/mm^3^), mean (SD)	412 (188.7)
Plasma HIV RNA (log_10_copies/ml), mean (SD)	5.62 (1.00)
**Baseline behaviour data**	*N* = 86
*Unprotected sexual intercourse infourmonths prior to study, n(%)*	72 (83.7)
With regular partner	55 (64.0)
With casual partner	49 (57.0)
With commercial sex worker	6 (7.0)
With person who gives money/goods	6 (7.0)
With IVDU partner	9 (10.5)
**Sexual partners in last four months (*n* = 86)**	
None	0 (0)
1	10 (11.6)
2–4	47 (54.7)
≥5	25 (29.1)
Decline to answer	4 (4.7)

^a^4th-generation (4thG) AHI stages: 4thG stage 1, NAT+/4thG immunoassay (IA)-/3rdG IA–; 4thG stage 2, NAT+/4thG IA+/3rdG IA–; 4thG stage 3, NAT+/4thG IA+/3rdG IA+/western blot– or indeterminate. MSM: men who have sex with men; SD: standard deviation; IVDU: intravenous drug user.

Any condomless sexual intercourse in the preceding four months was reported by 72 participants (83.7%). Condomless intercourse with regular partners, casual partners, commercial sex workers, paying partners and partners who inject drugs was reported by 55 (64%), 49 (57%), 6 (7%), 6 (7%) and 9 (10.5%) participants, respectively. None reported abstinence from sexual intercourse in the four months preceding the study while 10 (11.6%) reported a single partner. Two to four partners were reported by 47 participants (54.7%) and ≥5 partners by 25 (29.1%) participants. Four participants (4.7%) declined to answer.

**Figure 2.**
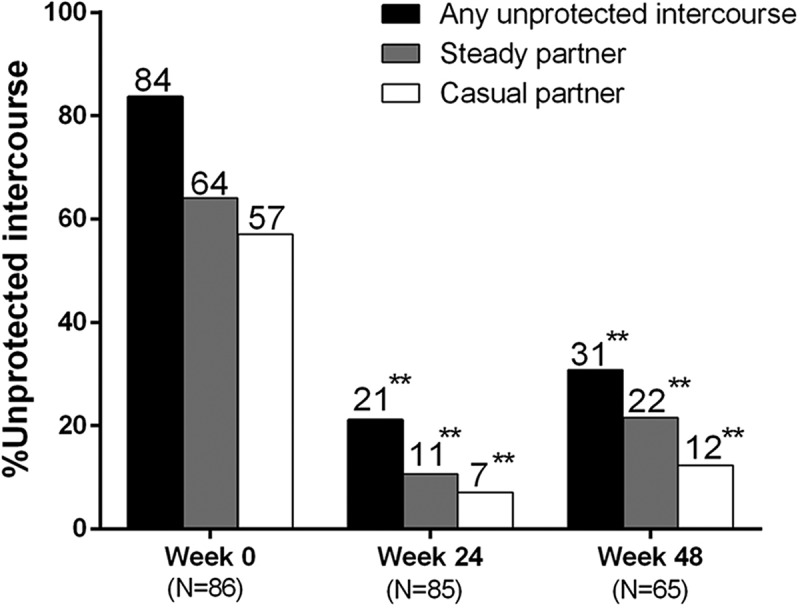
Percentage of participants reporting unprotected sexual intercourse in preceding four months. **p < 0.0001 vs. week 0

A large decrease in the number of sexual partners and condomless sex was reported following HIV diagnosis. At baseline, 72/86 (83.8%) reported multiple partners in the preceding four months, while 10/86 (11.6%) and 0/86 reported a single or no partners, respectively, and 4 (4.6%) declined to answer. This contrasts with 43/85 (50.6%, *p* < 0.001) and 29/65 (44.6%, *p* < 0.001) reporting a single partner or no partner at weeks 24 and 48, respectively. However, 25 (29.1%) of 86 participants reported ≥5 partners in the preceding four months at baseline while 9/65 (13.9%) reported ≥5 partners in the preceding four months at week 48 (*p* = 0.06); 4/9 participants were among the 25 who reported ≥5 partners at baseline. Any reported condomless sexual intercourse declined from 72/86 (83.7%) at baseline to 18/85 (21.2%) at week 24 (*p* < 0.0001) and 20/65 (30.8%) at week 48 (*p* < 0.0001) (Figure 2); 16/18 and 18/20 at weeks 24 and 48, respectively, were among the 72 who reported condomless sexual intercourse at baseline.

**Figure 3.**
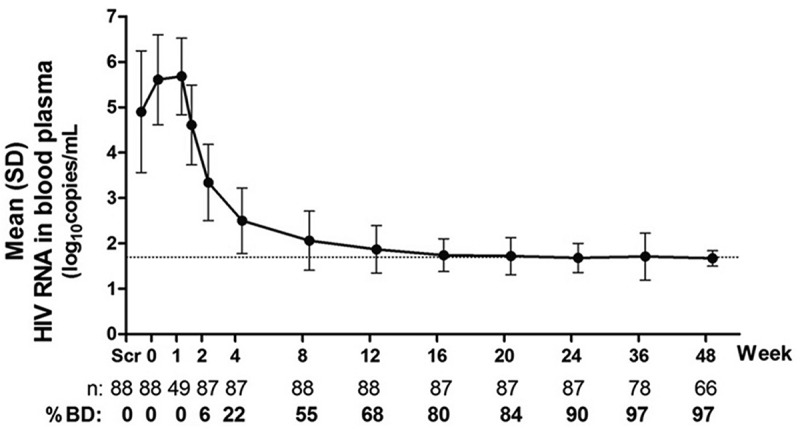
Plasma HIV RNA. BD: below detection (<50 copies/ml).

Mean plasma HIV RNA decline is shown in Figure 3; 5/87 (5.7%) participants attained HIV RNA <50 copies/ml by week 2, which increased to 48/88 (54.6%), 60/88 (68.2%), 78/87 (89.7%) and 64/66 (97%) at weeks 8, 12, 24 and 48, respectively.

### Modelling analyses

**Figure 4.**
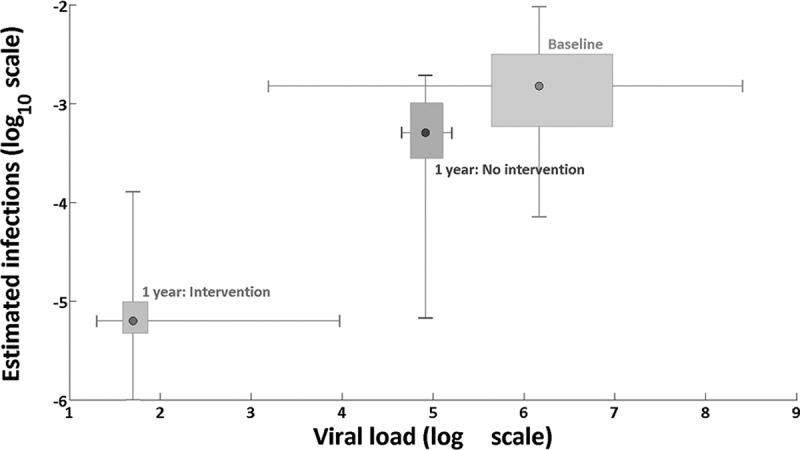
Estimated infections per infected individual per day.

**Figure 5.**
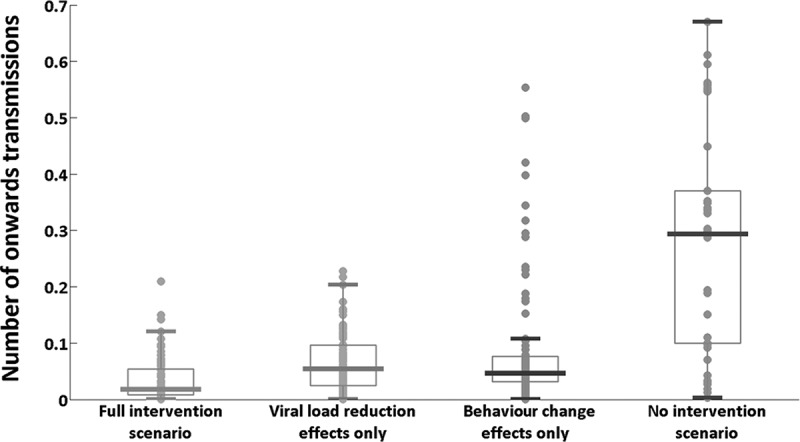
Estimated number of onwards HIV transmissions per infected individual during the first year of infection.

Based on viral load and transmission-related risk behaviour of each individual within the cohort, we estimated the rate of infectiousness per infected individual per day (Figure 4). Figure 5 illustrates the estimated number of onwards infections from each individual over the first year compared with expected number of transmissions without early diagnosis and ART.

**Figure 6.**
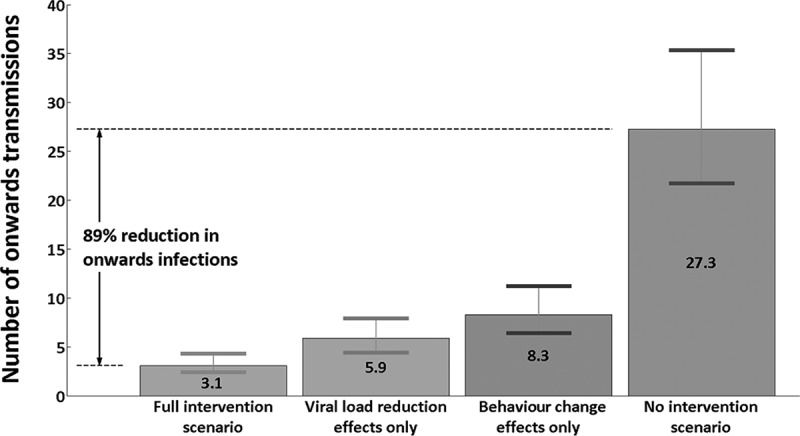
Estimated number of onwards transmissions across the cohort over one year of infection per participant.

In the baseline (actual) scenario, the mean number of estimated new infections caused per infected individual over their first year of infection was 0.035, whilst in the hypothetical scenario of no early detection and treatment this value was estimated at 0.294. In the hypothetical scenario where only viral load reduction is considered (without changes to risk behaviour), the mean number of estimated new infections caused per infected individual was 0.066. The value for the scenario in which only post-diagnosis behaviour changes are considered was 0.093. We also calculated potential additional second-, third- and fourth-generation transmissions in the first year from the index cases in the cohort. In the baseline scenario, an estimated mean 3.00 (95% credible interval: 2.31–4.18) direct infections and 0.08 (0.06–0.11) indirect infections are caused by the cohort one year after HIV exposure, giving a total of 3.08 (2.37–4.29) new transmissions. In the hypothetical scenario of no early detection and treatment intervention, the estimated total number of new infections caused by the cohort after one year was 27.26 (21.68–35.26): 25.34 (20.09–32.85) direct and 1.92 (1.59–2.41) indirect transmissions. These results translate into an estimated 88.7% (83.8–91.1%) reduction in onwards transmissions, one year after HIV exposure due to early detection and treatment (Figure 6).

Under the scenario of post-diagnosis behaviour change only, an estimated 8.26 (6.39–11.24) transmissions are caused by the cohort (approximately 70% reduction compared to no intervention), whereas the number of new infections in the viral load reduction-only scenario was 5.88 (4.42–7.91) (approximately 78% reduction compared to no intervention).

## Discussion

Our study provides evidence of substantial impact in reducing HIV transmission among Thai MSM through diagnosing HIV during acute infection and early initiation of ART. Post-diagnosis behavioural changes can significantly reduce the number of new HIV infections, but viral load reductions through ART have the greatest impact and together are estimated to reduce transmission by 89% over the first year of infection.

The broader population impact of such interventions may depend on the stage of the HIV epidemic. Current literature estimates the fraction of HIV transmissions in urban MSM populations resulting from AHI partners to be anywhere from 4% to 50% [13,16,19,40–45]. The phylogenetic approach used by Brenner and colleagues in the Quebec PHI Cohort leads to the higher estimate of early infection accounting for approximately half of onwards transmissions [13]. This finding is supported by phylogenetic studies in MSM populations elsewhere and would fit the ongoing incidence in MSM in Bangkok with a reported 60-month cumulative HIV incidence of 23.9% (31.3% for 18–21-year-olds) and overall HIV incidence of 5.9 per 100 person years [17]. It is reasonable to expect that in such a rising and ongoing HIV epidemic, AHI detection and control may be a powerful HIV prevention measure. We also demonstrated that AHI detection and early ART initiation are feasible to implement.

Benefits of treatment as prevention for MSM are considered highly plausible [1,46]. However, prevention benefits may be attenuated by other factors such as increasing condomless sex or STIs. The ongoing PARTNER and Opposites Attract studies provide the strongest evidence to date that treatment as prevention is indeed effective in MSM, with no transmissions reported to date in the PARTNER study among the sero-discordant MSM couples in whom the HIV-positive partner had HIV RNA <200 copies/ml [47,48]. The study’s analysis estimated within-couple risk of HIV transmission for condomless receptive anal intercourse when ejaculation occurred to be 0 (zero) per 100 couple years (upper 95% confidence limit 2.7) despite an STI incidence of 17–18% among the participating MSM. The benefit of treatment as prevention not being negatively impacted by condomless sex or STI in these studies, yet a high HIV incidence in some MSM communities with high ART uptake, would fit with AHI being an important factor in transmission in these communities [47]. If so, then to meet the UNAIDS target of eliminating HIV by 2030, AHI detection and treatment would need to be prioritized [49]. To the best of our knowledge, there are no studies specifically looking into treatment as prevention which target people during AHI. Our study therefore adds to the current literature by offering a first estimate of its possible impact based on actually observed AHI treatment effects.

The public health challenges of optimizing the engagement of care cascade to facilitate treatment as prevention strategies at the population level have been well described, recently with focus on engaging people with AHI [50,51]. While identifying AHI individuals remains challenging, AHI screening yield clearly depends on HIV prevalence in a given population and can be cost-effectively improved by adding pooled NAT testing to diagnostic algorithms for populations at high risk [32,52,53]. AHI incidence at the Thai Red Cross Anonymous Clinic was previously reported at 2.2 per 100 person years but seems to be rising after successfully engaging more MSM in HIV testing and counselling through Adam’s Love, an innovative web-based communications strategy. Since its introduction, the annual number of participants identified with AHI has increased from 25 to over 100 at this clinic [54]. Our study adds to a growing body of evidence that once identified, AHI may present a unique opportunity for behavioural counselling and entry into care [55–60]. We observed a significant reduction in sexual partners and a significant increase in condom use among study participants as reported at 24-week follow-up on study which was sustained throughout week 48, but our data cannot establish whether this was impacted by the behavioural counselling provided in the study or might be seen based on awareness of diagnosis alone [56–58].

While we need longer follow-up data to assert whether the observed behavioural changes are sustained, it is very encouraging that they are at least sustained until the vast majority of participants have reached plasma HIV RNA below the level of detection. Screening for AHI and linkage to care and treatment have been routinely practised in other settings, including North America and Australia, showing both feasibility and cost efficacy [61–66].

Our study is subject to limitations, which should be noted in interpreting results. The main finding of estimated transmissions averted is dependent on the relationship between viral load and HIV transmission probabilities. The relationship used was derived from heterosexual discordant couples in latent infection but adjusted to reflect higher transmission probability magnitudes for MSM in Bangkok and calibrated to local incidence and prevalence data. Calculations used numbers of self-reported transmission-related risk events, which are subject to potential bias. Social desirability and information bias cannot be excluded in gathering historical information on sexual behaviour. Given the sensitive nature of the information, we assume that self-reported behavioural data may underestimate number of partners and overestimate condom use. Standardized medical history taking at each follow-up visit readdressed these questions as a result of which such phenomena were minimized as much as possible. The duration of time from infection to diagnosis cannot be verified or estimated precisely, regardless of evidence of acute HIV detection among the individuals included in this study.

## Conclusions

There is strong evidence that ART not only has individual clinical benefits but also substantially reduces transmission risk. Given the large contribution of AHI to population incidence, we aimed to assess the potential effect of targeting detection of AHI in Bangkok-based MSM and initiation of early ART. We estimated that this approach could lead to 89% reduction in HIV transmissions during the first year of HIV infection when levels of transmission are highest. We believe that global elimination targets by 2030 require investment in prioritizing detection and treatment of early HIV infection. We have provided evidence that intensive efforts with such a strategy can lead to large changes in transmission and thus potential epidemic reversal among MSM.
